# Clinical Utilization and Cost of Thrombophilia Testing in Patients with Venous Thromboembolism

**DOI:** 10.1055/s-0040-1714334

**Published:** 2020-08-09

**Authors:** Manila Gaddh, En Cheng, Maha A.T. Elsebaie, Imre Bodó

**Affiliations:** 1 Department of Hematology and Medical Oncology, Emory University School of Medicine, Atlanta, Georgia, United States; 2 Department of Chronic Disease Epidemiology, Yale University School of Public Health, New Haven, Connecticut, United States; 3 Department of Medicine and Hematology, Semmelweis University, Budapest, Hungary

**Keywords:** venous thromboembolism, venous thrombosis, costs and cost analysis, thrombophilia, hypercoagulability

## Abstract

**Introduction** Testing for inherited and acquired thrombophilias adds to the cost of care of patients with venous thromboembolism (VTE), though results may not influence patient management.

**Methods** This is a single-center, retrospective study conducted at Emory University Hospitals from January to December 2015 to (1) determine the pattern of thrombophilia testing in patients with VTE, (2) study the impact of results of thrombophilia testing on clinical decision-making, and (3) determine the direct costs of thrombophilia testing in patients with VTE.

**Results** Of the 266 eligible patients, 189 (71%) underwent testing; 51 (26.9%) tested positive and the results impacted management in 32 (16.9%) of tested patients. Patient undergoing testing were more likely to be younger than 40 years (30.9 vs. 18.2%), have had prior pregnancy loss (9.0 vs. 0%), or known family history of hypercoagulability (24.9 vs. 10.4%), and were less likely to have had provoked VTE (37 vs. 79.2%). The most common thrombophilias tested were antiphospholipid syndrome (60.1%), factor V Leiden (59.7%), and prothrombin gene mutation (57.5%). Direct costs of thrombophilia testing were $2,364.32 per patient, $12,331.55 to diagnose 1 positive, and $19,653.41 per patient-management affected.

**Conclusion** We noted significant variability in selection of patients and panel of tests, sparse utilization of test results in patient management, but high cost associated with thrombophilia testing in patients with VTE. With guidelines advocating selective use of thrombophilia testing and attention to potential impact of test results in patient management, we propose the need for measures at institutional levels to improve test-ordering practices.

## Introduction

Since the discovery of antithrombin (AT) deficiency as an inherited thrombophilia in 1965, several inherited and acquired thrombophilias have been described as risk factors for venous thromboembolism (VTE). 1 As far as VTE management is concerned, the role of thrombophilias in determining the duration or choice of anticoagulant remains uncertain. 2 3 In everyday practice, however, physicians and patients are often inclined to request thrombophilia testing in the hope of (1) finding a predisposing cause for VTE, (2) understanding the patients' risk of VTE recurrence, (3) estimating VTE risk for family members, and (4) obtaining information that would help optimize management.

There is no defined panel of thrombophilia testing endorsed by guidelines. 4 Moreover, physicians are directed to determine duration of anticoagulation for an individual patient based on an assessment of the patient's risk for recurrent VTE and bleeding. 5 British and National Institute for Health and Care Excellence guidelines go on to suggest using thrombophilia testing only if it is determined that the results will impact patient management. 6 There is considerable heterogeneity in the relative risk of recurrence associated with individual thrombophilias reported in literature. 7 8 Determining the role of thrombophilia itself in the occurrence or recurrence of VTE in an individual patient is further complicated by the fact that multiple intrinsic and situational factors such as age, gender, body mass index, pregnancy, and postoperative state may interact variably with the underlying thrombophilia to manifest a thrombotic event. Limited data exist on the comparative effectiveness of different classes of anticoagulants in patients with underlying thrombophilia. A recent systematic review and meta-analysis suggests superiority of vitamin K antagonists over direct oral anticoagulants (DOACs) in patients with high-risk antiphospholipid syndrome (APS), while reporting equivalent efficacy and safety of these treatment options in the rest of the thrombophilias. 9 10

In these circumstances, lack of specific guidance from academic societies regarding adaptation of thrombophilia testing in clinical practice can lead to significant variability in what tests are ordered, when they are ordered, and how they are interpreted. While the results may not add value to patient management, the tests certainly increase the cost of management of venous thromboembolic disorders. 11

We performed this study to explore the pattern of thrombophilia testing, impact of the thrombophilia workup results on clinical management decisions, and direct cost of such tests in patients with VTE at our tertiary care center.

## Materials and Methods

### Study Design

This is a single-center, retrospective study conducted at Emory University Hospitals with the following objectives: (1) determine the pattern of thrombophilia testing in patients with VTE, (2) study the impact of results of thrombophilia testing on clinical decision-making, and (3) determine the direct costs of thrombophilia testing in patients with VTE. The study was approved and a waiver of patient informed consent was granted by Emory University Institutional Review Board (IRB).

### Patient Identification

The Hematology Service at Emory University Hospitals maintains an IRB-approved database for all patients seen by the Hematology Service in the inpatient or outpatient settings. From the Emory Hematology Service database, we identified adult patients, who were seen by Emory Hematology for the evaluation and treatment of VTE between January and December 2015 in the inpatient or outpatient settings. Exclusion criteria included: (1) no formal evaluation by the Emory Hematology Service (e.g., patients never showed up to any of their appointments), (2) insufficient information on VTE event in patient chart, (3) no history of VTE, or (4) superficial venous thrombosis only.

### Data Extraction

For eligible patients, electronic medical records (EMRs) were reviewed for data related to sociodemographics, medical history, details of thromboembolic events, thrombophilia workup, and patient management ( Table 1 ). Patient data was extracted manually into predesigned case report forms, then deidentified and anonymized prior to analysis.

**Table 1:** Data collected in case report forms

Demographic data	Age at time of evaluation Gender, race Height, weight, and BMI
Clinical data	Location of latest VTE Thromboembolic risk factors present History of VTE or pregnancy losses History of VTE or early-age stroke/MI (< 50 y) in 1st degree relatives Known hypercoagulable state in family Comorbidities present
Thrombophilia workup	Number and result of thrombophilia tests Antiphospholipid antibodies a Factor V Leiden Prothrombin G20210A mutation Antithrombin deficiency Protein S deficiency Protein C deficiency Jak 2 mutation Paroxysmal nocturnal hemoglobinuria Others Repeat tests and their results Reasons of incomplete workup
Management data	Diagnostic workup for VTE Management plan, and any changes influenced by thrombophilia results
Institutional data	Cost of tests

Abbreviations: BMI, body mass index; MI, myocardial infarction; VTE, venous thromboembolism.

a Lupus anticoagulant, immunoglobulin (Ig) G and IgM anticardiolipin, IgG and IgM anti-β2 glycoprotein I.

We categorized the most recent VTE event at the time of initial hematology consult as our index episode. The following VTE episodes were classified as “provoked” per the International Society of Thrombosis and Haemostasis definition: (1) VTE occurring within 3 months of surgery with general anesthesia, cesarean section, prolonged hospital stay, estrogen therapy, or prolonged immobility, and (2) VTE associated with an indwelling venous catheter or underlying cancer. 12

### Thrombophilia Testing

All tests ordered by treating physicians as part of thrombophilia workup were included for description of the pattern of thrombophilia testing. Thrombophilia workup was defined as complete if it included tests for common inherited and acquired thrombophilias that are known to have a reasonably well-defined role in pathogenesis of VTE based on contemporary knowledge. These include factor V Leiden (FVL), prothrombin G20210A (PT) gene mutation, AT activity level, protein S (PS) activity level, protein C (PC) activity level, and antiphospholipid antibody (APLA) panel. The APLA panel comprised of lupus anticoagulant, immunoglobulin (Ig) G and IgM anticardiolipin antibody, and IgG and IgM anti-β2 glycoprotein I antibody.

“Incomplete workup” included (1) Failure to order any of the aforementioned tests. (2) “Wrong timing”: testing for PC, PS, and AT activity levels under conditions that might have jeopardized accurate interpretation of results: testing within 1 week of acute thrombosis or during ongoing anticoagulation with warfarin for PC and PS activity levels, heparin for AT activity level, and DOACs for PC, PS, and AT activity levels. 4 13 14 (3) Failure to repeat a positive APLA panel after 12 weeks of initially positive test results. 13 14 Functional PC, PS, and AT assays were called abnormal if they were out of the normal laboratory ranges. Given that establishing a diagnosis of natural anticoagulant deficiency requires confirmatory repeat testing, physician's assessment, and correlation with the patient's clinical history, the final diagnosis (e.g., PS deficiency) was based on the interpretation of results by the treating hematologist.

Repeated functional/antigenic assays were categorized as: (1) Appropriate/justified, if assays were repeated to confirm initial abnormal results, or to account for “wrong timing” and uninterpretable initial test results. (2) Inappropriate/unjustified, if initial assays were normal, or if assays were repeated during “wrong timing” as specified above. Repetition of any genetic testing was considered unjustified. Criteria for appropriateness of test repeats were based on the recommendations in the review articles by Nakashima and Rogers, Tientadakul et al, and Mahajerin et al. 13 15 16 Given the uncertainty about the role of monitoring APLA titers in patients with established APS, we did not include the APLA panel in our analysis of appropriateness of repeated testing.

The influence of thrombophilia testing results on patient management was measured as follows: (1) effect on choice on anticoagulant, and (2) effect on duration of anticoagulation. Information on change in patient management was obtained from physician notes that followed thrombophilia testing.

All tests ordered as part of thrombophilia evaluation were included in the calculation of direct cost of the workup. The laboratory charges associated with individual thrombophilia tests were obtained from the Emory University pathology laboratory services and was based on the year 2015 rates ( Table 2 ). For each thrombophilia test, the tested patients were assigned a cost value that reflected the laboratory charges for the thrombophilia test multiplied by the test-ordering frequency. For patients who were tested outside the Emory Healthcare System, and for whom we were unable to verify the test-ordering frequency, we made the assumption that they were tested once. One exception was patients who had been given a diagnosis of APS. For such patients, the frequency of ordering the APLA panel was considered as two.

**Table 2:** Direct medical costs of thrombophilia testing at Emory University Hospitals (in US dollars)

Test name	*N* tested patients	Mean cost a	Minimum cost	Maximum cost b
Antiphospholipid antibody (APLA) panel	160	$2,846.03	$553.00	$15,398.00
Factor V Leiden	159	$119.44	$75.00	$648.00
Prothrombin mutation	153	$86.59	$44.00	$522.00
Antithrombin level c	151	$134.98	$54.13	$408.00
Protein S level c	133	$182.41	$37.00	$1,062.00
Protein C level c	127	$160.78	$65.35	$480.00
Jak 2 mutation	25	$174.41	$80.00	$324.00
PNH flow cytometry	22	$1,168.45	$138.00	$2,005.00
Fibrinogen activity level	77	$89.79	$30.00	$104.00
Homocysteine level	72	$139.41	$60.00	$167.00
Factor VIII level	55	$207.45	$63.00	$204.00
Factor IX level	48	$214.28	$67.00	$216.00
Factor XI level	47	$202.35	$63.00	$204.00
Lipoprotein a level	33	$32.76	$33.00	$46.00
MTHFR mutation	2	$119.00	$119.00	$119.00

Abbreviations: MTHFR, methylenetetrahydrofolate reductase; PNH, paroxysmal nocturnal hemoglobinuria.

Note: Direct medical costs reflect the costs of the testing kit, test-ordering frequency, and any other laboratory costs.

a Mean cost = Total cost of testing for each corresponding thrombophilia/Total *N* of patients tested for this thrombophilia.

b Maximum cost reflects the patient who had the highest cost value in each corresponding test. A high cost value could reflect using a more expensive testing kit (e.g., factor V Leiden [FVL] tested in clinic [$216.00] vs. hospital [$75.00]), and/or frequent testing that multiplied the total cost for this corresponding thrombophilia.

c The corresponding cost combines the cost of antigen and activity level testing.

*Statistical analysis* : We used the chi-square tests for categorical variables and the Kruskal–Wallis test for continuous variables to compare the distribution of variables between tested and nontested patient groups. Costs of thrombophilia workup were summarized using descriptive statistics. All analyses were conducted using SAS, version 9.4 (SAS Institute, Inc, Cary, North Carolina, United States), and SPSS, version 25.0 (IBM SPSS statistics). A *p* -value of ≤ 0.05 was considered statistically significant.

## Results

**Fig. 1:**
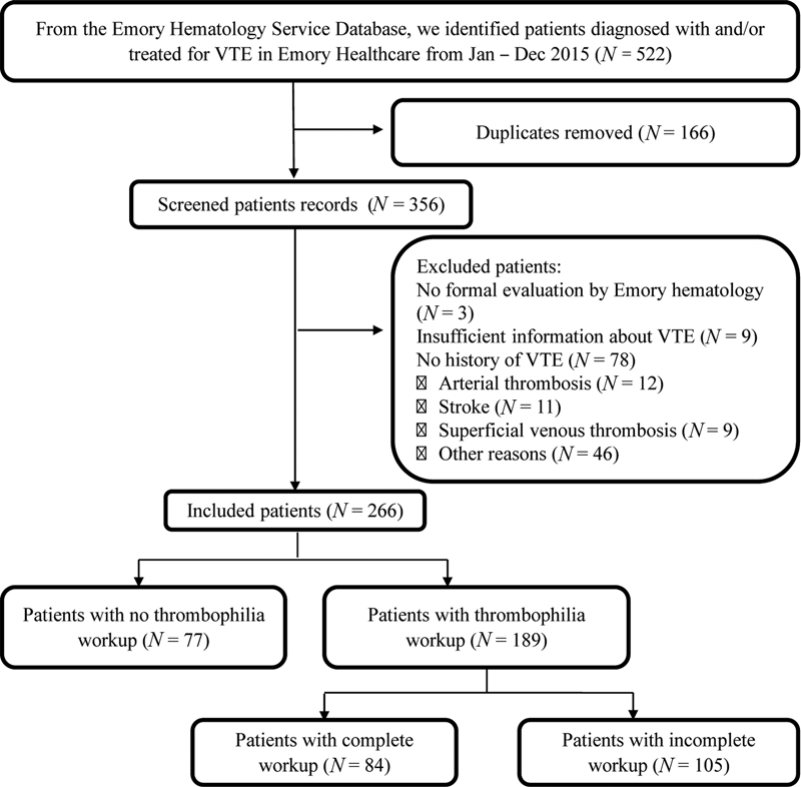
Flowchart for patient identification.

Hematology Service database for the year 2015 yielded 522 patients with a final diagnosis of VTE or hypercoagulable state. After excluding duplicates and patients who did not meet the inclusion criteria, 266 eligible patients were identified ( Fig. 1 ).

Patient characteristics are presented in Table 3 . Of the 266 included patients, 189 (71.1%) underwent thrombophilia workup. The cohort of patients that underwent thrombophilia testing was more likely to be younger than 40 years (30.9 vs. 18.2%), have had prior pregnancy loss (9.0 vs. 0%), or known family history of VTE/thrombophilia/early age stroke/myocardial infarction (24.9 vs. 10.4%). This cohort was less likely to have had provoked VTE (37 vs. 79.2%), particularly VTE associated with preceding surgery (9.5 vs. 31.2%), indwelling venous catheter (7.4 vs. 18.2%), or active malignancy (5.8 vs. 41.6%). There were no statistically significant differences between the tested and nontested groups in gender, racial distribution, pregnancy, hormonal use, location of clot (visceral vs. nonvisceral), or history of prior VTE. Overall, 53% of patients with provoked VTE underwent thrombophilia testing.

**Table 3:** Patient characteristics

Characteristics	Patients with thrombophilia workup ( *N* = 189)	Patients without thrombophilia workup ( *N* = 77)	*p* -Value
Age at evaluation; median (range)	52 (19–88)	61 (20–85)	**< 0.001**
Age at first VTE; median (range)	50 (14–87)	59 (20–85)	**< 0.001**
Age at first VTE < 40 y	58 (30.9)	14 (18.2)	**0.035**
Sex			
Male	86 (45.5)	43 (55.8)	0.126
BMI; median (range)	29.7 (14.2–58.7)	28.7 (13.5–55.0)	0.213
Race			
White	92 (48.7)	43 (55.8)	0.437
African American	77 (40.7)	29 (37.7)	
Other a	20 (10.6)	5 (6.5)	
Provoked VTE b	70 (37.0)	61 (79.2)	**< 0.001**
Preceding surgery	18 (9.5)	24 (31.2)	**< 0.001**
Preceding nonsurgical hospitalization/ immobilization c	23 (12.2)	9 (11.7)	0.913
Hormone associated (estrogen therapy)	15 (7.9)	6 (7.8)	0.384
Pregnancy/Puerperium associated	6 (3.2)	1 (1.3)	0.254
Indwelling venous catheter associated d	14 (7.4)	14 (18.2)	**0.009**
Prior VTE	62 (32.8)	21 (27.3)	0.377
Prior pregnancy loss(es)	17 (9.0)	0 (0.0)	**0.003**
High risk comorbidities			
Active malignancy	11 (5.8)	32 (41.6)	**< 0.001**
Chronic inflammatory disorders e	13 (6.9)	2 (2.6)	0.170
Nephrotic syndrome	3 (1.6)	0 (0.0)	0.266
Congestive heart failure	8 (4.2)	7 (9.1)	0.119
Stroke/TIA	11 (5.8)	1 (1.3)	0.107
Family history of VTE, early age stroke/MI or known thrombophilia	47 (24.9)	8 (10.4)	**0.008**
Most recent VTE location			
Visceral DVT	32 (16.9)	7 (9.1)	0.101 f
Nonvisceral			
Lower extremity DVT only	52 (27.5)	29 (37.7)	
Upper extremity DVT only	8 (4.2)	13 (16.9)	
Pulmonary embolism only	55 (29.1)	15 (19.5)	
> 1 clot	51 (27.0)	15 (19.5)	

Abbreviations: DVT, deep venous thrombosis; MI, myocardial infarction; TIA, transient ischemic attack; VTE, venous thromboembolism.

Note: Categorical variables were presented as *N* (%). Bold values reflect significant *p* < 0.1.

a Other races include Asian, and Hispanic, and no available race.

b Transient factors do not include active malignancy, chronic inflammatory conditions, or chronic infections.

c Includes fractures leading to immobilization for at least 3 days.

d Catheter associated includes VTE associated with central line, left ventricular assist device, pacemaker, or implantable cardioverter-defibrillator.

e Chronic inflammatory disorders include inflammatory bowel disease, systemic lupus erythematosus, and vasculitis.

f *p* -Value for visceral versus nonvisceral clot locations.

**Fig. 2:**
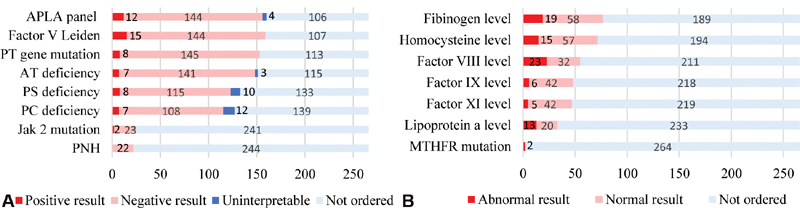
Results of thrombophilia workup in all patients ( *N* ). ( **A** ) Thrombophilias with reasonably well-defined role in pathogenesis of venous thromboembolism (VTE) based on contemporary knowledge. Uninterpretable APLA testing indicates abnormal APLA panels that were not repeated. Uninterpretable AT, PS, and PC tests include tests that were performed under conditions that jeopardized accurate interpretation of results such as acute thrombosis or ongoing anticoagulation. ( **B** ) Other thrombophilias. APLA, antiphospholipid antibody; AT, antithrombin. MTHFR, methylenetetrahydrofolate reductase; PC, protein C; PNH, paroxysmal nocturnal hemoglobinuria; PS, protein S; PT, prothrombin.

The pattern of thrombophilia workup, including details of results, is presented in Fig. 2 . The most common thrombophilias tested for were APS in 160 (60.1%), followed closely by FVL in 159 (59.7%) and PT gene mutation in 153 (57.5%) patients. Of the 189 tested patients, 84 (44.4%) fulfilled the criteria for complete thrombophilia workup. Of patients with abnormal functional assays, 9 were tested during the acute thrombosis period and 13 were tested while receiving therapeutic anticoagulation rendering their results uninterpretable. Four patients with positive APLA panel did not undergo repeat testing to determine persistence. Fifty-one (26.9%) patients tested positive for one or more of the studied thrombophilias.

**Fig. 3:**
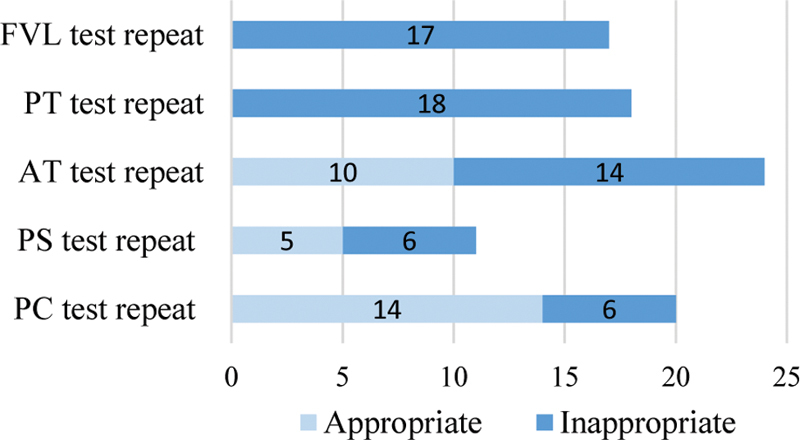
Appropriateness of thrombophilia test repeats ( *N* ). AT, antithrombin; FVL, factor V Leiden; PC, protein C; PS, protein S; PT, prothrombin.

Table 4 presents the pattern of repeat testing for the tests included in the “complete thrombophilia” panel. Among the 159 patients tested for FVL and the 153 patients tested for PT G20210 mutation, genetic testing was repeated in 17 (10.7%) and 18 (11.8%) patients, respectively. Nine patients were tested for a third time. There was no significant correlation between the initial genetic test result and decision to repeat testing. On the other hand, the AT/PS/PC test repeats occurred more frequently in response to an abnormal (deficient/uninterpretable) rather than a normal initial test result. Of the 83 patients found to have abnormal AT/PS/PC assays, 31 (37.3%) had repeat testing for confirmation. Seven patients were tested three times. From all the testing performed, hereditary AT/PS/PC deficiency was eventually diagnosed in 22 cases. The appropriateness of thrombophilia test repeats as determined by our predefined criteria is illustrated in Fig. 3 . Twenty-six of the 55 repeated AT/PS/PC assays were deemed inappropriate because initial assays were normal or assays were repeated during “wrong timing” per criteria described in the “Methods” section.

**Table 4:** Pattern of repeat thrombophilia testing

First test results	*N*	Was test repeated?			Number of test repeats			
		Yes	No		2nd test	Positive result	3rd test	Positive result
		*N* (%)	*N* (%)	*p-* Value	*N*	*N* (%)	*N*	*N* (%)
FVL								
Positive	15	2 (13.3)	13 (86.7)	0.728	2		0	
Negative	144	15 (10.4)	129 (89.6)		12		3	
Total	159	17 (10.7)	142 (89.3)		14	2/14 (14.3%)	3	0/3 (0.0%)
PT mutation								
Positive	8	1 (12.5)	7 (87.5)	0.947	1		0	
Negative	145	17 (11.7)	128 (88.3)		11		6	
Total	153	18 (11.8)	135 (88.2)		12	1/12 (8.3%)	6	0/6 (0.0%)
AT level								
Deficient a /Uninterpretable	20	10 (50.0)	10 (50.0)	**< 0.001**	10		0	
Normal	131	14 (10.7)	117 (89.3)		8		6	
Total	151	24 (15.9)	127 (84.1)		18	2/18 (11.1%)	6	0/6 (0.0%)
PS level								
Deficient b /Uninterpretable	25	6 (24.0)	19 (76.0)	**0.002**	3		3	
Normal	108	5 (4.6)	103 (95.4)		4		1	
Total	133	11 (8.3)	122 (91.7)		7	1/7 (14.3%)	4	1/4 (25.0%)
PC level								
Deficient c /Uninterpretable	38	15 (39.5)	23 (60.5)	**< 0.001**	11		4	
Normal	89	5 (5.6)	84 (94.4)		4		1	
Total	127	20 (15.7)	107 (84.3)		15	1/15 (6.7%)	5	2/5 (40.0%)
Jak 2 mutation								
Positive	2	0	2 (100.0)	N/A	0		0	
Negative	23	0	23 (100.0)		0		0	
Total	25	0	25 (100.0)		0	N/A	0	N/A
PNH flow cytometry								
Positive	0	0	0 (100.0)	N/A	0		0	
Negative	22	0	22 (100.0)		0		0	
Total	22	0	22 (100.0)		0	N/A	0	N/A

Abbreviations: AT, antithrombin; FVL, factor V Leiden; N/A, not applicable; PC, protein C; PNH, paroxysmal nocturnal hemoglobinuria; PS, protein S; PT, prothrombin.

Notes: AT/PS/PC antigen and activity levels were called “deficient” if they were below the low normal value in the reference laboratory range. Bold values reflect significant *p* < 0.1.

a The normal range for AT antigen level was 214–318, and the normal range for AT activity level was 83–128%.

b The normal range for PS antigen level was 70–155, and the normal range for PS activity level was 64–149%.

c The normal range for PC antigen level was 70–140, and the normal range for PC activity level was 90–183%.

Thrombophilia testing influenced patient management in 32 (16.9%) of the 189 tested patients ( Table 5 ). Results of thrombophilia testing influenced choice of anticoagulant in 6, duration of anticoagulation in 25, and both choice and duration of anticoagulation in 1 patient (details provided in footnotes of Table 5 ). Clinical management was modified in 7 of the 12 patients with positive APLA in our study; Warfarin was chosen over DOACs in four patients, and anticoagulation was extended indefinitely in the other three patients. In 19 patients who had negative workup or tested positive only for a low-risk thrombophilia (e.g., heterozygous FVL), physicians felt encouraged to carry out a safe discontinuation trial. The outcome of this trial was guided by the D-dimer levels measured 1 month after discontinuation of anticoagulation therapy. Of the remaining patients (83.1%) whose management was not affected by thrombophilia testing, 6 were lost to follow-up and 2 refused further management changes. Anticoagulation therapy was extended in 12 patients despite negative thrombophilia workup because of high D-dimer levels, high residual clot burden, persistent symptoms, or persistent risk factors.

**Table 5:** Effect of thrombophilia workup on management

Was management plan changed? *N* (%)		How was management plan changed?		
		Choice of AC, *N* (%)	Duration of AC, *N* (%)	Choice and duration of AC, *N* (%)
Yes	32/189 a (16.9)	6/32 b (18.8)	25/32 c (78.1)	1/32 d (3.1)
No	157/189 (83.1)	N/A	N/A	N/A

Abbreviations: AC, anticoagulation; APS, antiphospholipid syndrome; AT, antithrombin; FVL, factor V Leiden; N/A, not applicable; PS, protein S.

a 189 represent total number of patients who underwent any thrombophilia workup.

b Three patients with negative antiphospholipid antibody (APLA) panel were switched from warfarin to direct oral anticoagulants. In another three patients, warfarin was continued/started because of positive APS workup. One patient with heterozygous FVL and 18 patients with negative workup were taken off anticoagulation, after a safe discontinuation trial.

c Anticoagulation was extended in three patients with a positive APS antibody panel, one patient with PS deficiency, one patient with elevated factor VIII and IX, and one patient with AT deficiency.

d In one patient with a triple positive APLA panel, anticoagulation was switched to warfarin and continued indefinitely.

In our cohort of 266 patients, we calculated that the direct cost of thrombophilia testing was $2,364.32 per patient, $12,331.55 to diagnose one positive case, and $19,653.41 per patient-management affected. This amounted to a total annual expenditure of $628,909.12 toward the direct cost of thrombophilia testing. Table 6 presents the detailed direct costs of thrombophilia workup in the entire cohort as well as in the patient subgroup that underwent thrombophilia workup.

**Table 6:** Cost of thrombophilia workup (US dollar)

	Cost per patient	Cost per positive patient
All patients ( *N* = 266)	2,364.32	12,331.55
Patients with workup ( *N* = 189)	3,308.43	12,260.65
Patients with incomplete workup ( *N* = 105)	2,701.69	12,894.43
Patients with complete workup ( *N* = 84)	4,066.84	11,779.81

Note: Cost per patient = total cost/total *N* of patients. Cost per positive patient = total cost/total *N* of positive patients.

## Discussion

We have presented the pattern and clinical utilization of thrombophilia testing, and the direct cost associated with such tests at our institution. We noticed significant variability in selection of patients for thrombophilia testing, and in the panel of tests ordered in individual patients. Although the direct cost of testing was high, results impacted management decisions in very few patients.

The question of who should be tested for thrombophilia remains a matter of heightened debate. While there are no clinical trials available to provide evidence-based guidance on the issue, consensus guidelines from national and international academic societies recommend against indiscriminate testing for hereditary/acquired thrombophilia in patients with VTE. 2 17 18 A significant proportion of patients in our cohort underwent thrombophilia testing, with younger patients, those with unprovoked thrombosis, known family history of thrombosis or thrombophilia, or without a personal history of cancer being more likely to have undergone testing. The patient selection in our cohort seems to follow the “testing selectivity” recommended by some guidelines. For example, the British Society of Haematology recommends testing in patients who present with VTE at an early age (< 40 years old), recurrent VTE, or with family history of unprovoked thrombosis in 1st degree relatives. 18 Nonetheless, there was a noticeable proportion of patients in our cohort that underwent thrombophilia testing in the absence of any of the aforementioned indications. Fifty-three percent of patients with provoked VTE underwent thrombophilia testing in our study, corresponding to 37% of total tested patients.

It is well known that the relative risk for VTE associated with different clinical thromboembolic provoking factors is variable. Surgical hospitalization, for example, is associated with a significantly increased risk for VTE (odds ratio = 18.95, 95% confidence interval 9.22–38.97), whereas estrogen use increases this risk by only 1.81 times. The latter is, therefore, considered a “weak” clinical risk factor. 4 19 20 This could explain, in part, some of the testing decisions among patients with provoked VTE in our cohort. Similar pattern was reported in a large cohort of 1,314 patients with VTE by Meyer et al; the presence of “weak” thromboembolic risk factors (e.g., pregnancy or hormone use) did not seem to affect the decision of thrombophilia testing, whereas active cancer, recent hospitalization, lung disease, or indwelling venous catheters were significantly associated with no thrombophilia testing. 21

Unnecessary or unreliable thrombophilia testing has been reported by many researchers, and reflects the persistent problem of poor patient and timing selection. 21 22 23 In our cohort, 11.6% of all ordered tests were uninterpretable because of being confounded by acute clot or concurrent anticoagulation. Other researchers have observed even higher proportions of unreliable testing. In one study, 35.2% of patients were tested within 7 days of index VTE, and in another, 63% of abnormal PS and PC test results were attributed to concurrent anticoagulation therapy. 15 21 22 These observations raise concerns regarding the high likelihood of inaccurate results and their impact on the cost and management decisions for patients with VTE in our daily practice.

Thrombophilia testing is expensive, with many studies indicating a cost burden rather than a benefit of testing, because of lack of universal guidelines and the magnitude of inappropriate testing. 24 25 26 27 In our cohort of 266 patients, we calculated that the direct cost of thrombophilia testing was $2,364.32 per patient and $19,653.41 per one management change. These values do not account for the cost and complications downstream of extended anticoagulation in patients with potentially false positive results (i.e., lack of confirmatory repeat test) or for the anxiety of being diagnosed with a hereditary disorder. 28

Ideally, the decision to perform thrombophilia testing in patients with VTE should be based, in part, on whether results will influence clinical management. In our cohort, thrombophilia testing influenced the duration and/or choice of anticoagulation in only 16.9% of all tested patients. For VTE provoked by major temporary risk factors (e.g., surgical hospitalization), guidelines recommend a maximum of 3 months of therapeutic anticoagulation. 5 In patients with unprovoked VTE in whom extended anticoagulation is considered, the value of thrombophilia testing is rather controversial. It is important here to emphasize that a negative thrombophilia workup does not reduce the risk of VTE recurrence in patients with unprovoked VTE. 29 Therefore, deciding the duration of anticoagulation in these cases rests on the assessment of other pertinent factors such as patient characteristics, symptoms, D-dimer level, residual clot burden, and risk of bleeding. 30 One reasonable and frequently observed utility of thrombophilia testing in our patients with unprovoked VTE was to use the results to decide whether a discontinuation trial could be safe in patients who desired limited duration of anticoagulation or whose risk of bleeding was moderate.

As far as choice of anticoagulant is concerned, the only thrombophilia that has a bearing is high-risk APS (APS with triple antibody positivity and/or arterial thrombosis). 3 9 Recent studies have indicated that DOACs may be inferior to warfarin in high-risk APS patients due to a higher risk of arterial events with DOACs. 31 In our cohort, choice of anticoagulant was based on APLA results in seven patients; warfarin was chosen over DOACs in four because of positive results, and three patients were switched from warfarin to a DOAC after their APLA panels resulted negative. Therefore, there may be clinical utility for early testing for APS in patients with unprovoked VTE or VTE associated with “weak” clinical risk factors while making a decision about appropriate type of anticoagulant for individual patients.

Our study is limited mainly by its retrospective nature and reliance on chart documentation of relevant positive and negative histories. It is our standard hematology practice at Emory University Hospitals to include all pertinent positive medical history, including information on pregnancy losses, in the initial inpatient consult/outpatient visit note for patients who were referred for thrombosis. Patient information was collected from the initial visit note as well as notes from subsequent visits with Emory Hematology. Patients with insufficient information on the index VTE were excluded from the study ( *N* = 9, Fig. 1 ). This allowed completion of data to the best of our knowledge. For patients who received part of their thrombosis care outside Emory Healthcare, we were not able to verify the timing or the number of times a particular test was ordered for these patients. We reviewed EMR for patients who presented to the Emory Hematology service in 2015, and therefore, utilized the average charges for individual thrombophilia tests in the same year. In some cases, however, testing was done in preceding or following years, or outside of Emory Healthcare System, which may have undermined the precision of our calculated costs. Nevertheless, our results provide an estimate of total annual expenditure pertaining to thrombophilia testing and were close to that reported in other contemporary analyses. 28 While we realize that the cost of medical care including indirect costs and downstream effects of medical decisions have an impact on the overall cost-effectiveness of thrombophilia testing, this was beyond the scope of our inquiry.

## Conclusion

Our results highlight the variability in selection of patients and panel of tests for thrombophilia testing among patients with VTE. The direct cost associated with thrombophilia testing was high, though the results were utilized in clinical decision making in very few patients. With guidelines advocating selective use of thrombophilia testing and attention to potential impact of test results in patient management, we propose the need for measures at institutional levels to improve thrombophilia test-ordering practices. These strategies could include development of local guidelines, continuing medical education, and the implementation of clinical decision support systems within the electronic medical charts.
